# From the Laboratory to the Kitchen: New Alternatives to Healthier Bakery Products

**DOI:** 10.3390/foods8120660

**Published:** 2019-12-09

**Authors:** Miguel Peris, Susana Rubio-Arraez, María Luisa Castelló, María Dolores Ortolá

**Affiliations:** 1 Department of Chemistry, Universitat Politècnica de València, Camino de Vera, s/n. 46022 Valencia, Spain; 2 Institute of Food Engineering for Development, Universitat Politècnica de València, Camino de Vera, s/n. 46022 Valencia, Spain; suruar@upvnet.upv.es (S.R.-A.); mcasgo@upvnet.upv.es (M.L.C.); mdortola@tal.upv.es (M.D.O.)

**Keywords:** bakery, sweeteners, fat, protein, fiber

## Abstract

Due to the growing interest in improving the nutritional profile of bakery products, we have dealt with the most recent and relevant contributions regarding potential replacements for carbohydrates, proteins, and fats. Focusing on the influence of carbohydrates on metabolism, their excess implies obesity, diabetes and tooth decay. However, they are technologically important, since they are responsible for the structure of many bakery products. Regarding of the lipid profile, saturated fats have a great impact on the appearance of cardiovascular disease. Fortunately, nature and the food industry offer alternatives to traditional oils/butters with large amounts of omega 3 and other components that can mitigate these problems. Other relevant aspects are related to allergies concerning egg proteins, gluten or even requirements for vegan consumers. Several studies have been performed in this line, replacing eggs with milk serum, different mucilages obtained from legumes or some gums, etc. In conclusion, many papers have been published showing the possibility of successfully replacing (both at technological and sensory levels) less healthy ingredients with others that are nutritionally better. The challenge now is to combine these better components in a given product, as well as to evaluate possible interactions among them.

## 1. Introduction

The usual consumption of bakery products presents certain drawbacks related to their high content of simple sugars of rapid absorption, high fat content and low amount of dietary fiber, which make them highly caloric foods. Bakery products, including biscuits, cakes and bread, contain wheat flour as primary ingredient as it contributes to structure and volume [1,2,3]. According to recent data published by the World Health Organization (WHO) [4], 13% of the world’s adult population (11% of men and 15% of women) are clinically obese and 39% of adults aged 18 or older (40% of women and 38% of men) were found to be overweight. Regarding the child population, 41 million children under five are overweight or obese. Overweight and obesity are closely related to the increase in the intake of fat-rich high-calorie foods, as well as to the decrease in physical activity due to the progressively sedentary nature of many types of work, new transportation means and growing development [5]. 

In human nutrition, fat is an important ingredient in many foods used to improve product excellence [6,7]. Unfortunately, obesity and overweight are the main risk factors for noncommunicable illnesses, such as diabetes, cardiovascular diseases, and some types of cancer. Instead, the excessive consumption of simple sugars, along with their contribution to caloric intake, is related to the development of dental caries. Dental diseases are the most prevalent noncommunicable sicknesses in the world and their treatment consumes between 5% and 10% of healthcare budgets in industrialized countries [4]. Additionally, simple sugars of rapid absorption cause glycemic peaks and the excess sugar can rapidly become fat in the body [8,9,10]. 

Sucrose is a very important ingredient in bakery products for its conservation characteristics and a significant source of energy [11]. Moreover, there is a growing interest in replacing sucrose with alternative substances, such as low-calorie sweeteners (sucralose, tagatose, maltitol, stevia), in bakery products [12]. On the other hand, Trans Fatty Acids (TFA) are associated with an increased risk of coronary heart diseases, affecting inflammation factors and blood lipids [13]. 

Therefore, the World Health Organization, in its latest report [5], considers it urgent to carry out an immediate reduction in the extreme consumption of sugars and other fast-absorbing carbohydrates such as sucrose, as well as an increase in daily physical activity, to curb the tendency towards obesity and type 2 diabetes. Fortunately, consumers are well aware of the glycemic index (GI), caloric and dietary fiber content of foods. Furthermore, low GI diets have favorable effects on obesity-related sicknesses such as type 2 diabetes [14,15]. The consumer’s growing interest in healthier and tastier foods makes the food industry develop new bakery products [16]. Wholegrain foods are essential sources of dietary fiber and their ingestion has been associated with the prevention of chronic diseases due to their bioactive properties and health benefits [17,18]. In this sense, the aforementioned report also recommends the adoption of fiscal measures, such as a tax increase on certain food products and beverages that are rich in trans fatty acids, saturated fats, free sugars and/or salt, since an increase in the price of these products results in a decrease in their consumption. Nevertheless, for the food industry, and specifically the bakery sector, reducing the content of rapidly absorbed sugars along with a reduction in trans fatty acids and saturated fats is a great challenge if market share is to be maintained without breaking away from global and European policies.

Baking is the process that transforms dough in bakery products with exceptional sensorial features. Therefore, the aspect, color of the surface, and flavor of bakery products are the major qualities evaluated by consumers [19]. The bakery sector accounted for 10.7% of the total turnover of the food and drink industry in 2012. The sector encompasses more than 150,000 companies, representing 54% of the total number of firms in the drink and food industry [20]. Cakes or muffins, as well as cookies, are products consumed by all levels of society, due to their "ready to eat" format, their accessibility in different varieties, and their reasonable cost. However, the health benefits of these or other bakery products are questionable. Hence, its reformulation—by modifying the type of fats and carbohydrates occurring in them—would undoubtedly contribute to improving the quality of the product, and therefore the diet of its consumers; this also reduces the risk of cardiovascular diseases, as well as the appearance of dental caries or problems associated with obesity [21].

In view of the above, the purpose of the present work is to review the most recent studies dealing with the nutritional improvement of bakery products, carried out by replacing less healthy ingredients such as fats or sugars. Additionally, other papers related to the substitution of whey and/or egg proteins have also been mentioned, given the growing interest in products without this type of protein (intolerance problems).

## 2. Different Carbohydrates (Sweeteners) Used in the Bakery

Low-sugar or low-calorie labels are a top-ranked market trend for the bakery sector, mainly due to the fact that the overconsumption of sweets contributes to increasing obesity among children and adults, as well as other health problems. In this sense, the replacement of sugar with other natural sweeteners is a clear attempt to achieve a healthier lifestyle and has a major influence on the proposals of the bakery industry in terms of the development of innovative products. The market aims at removing the unhealthy ingredients in formulations, particularly sugars, but pays attention to customer satisfaction. Baked goods manufacturers are currently utilizing intense as well as high-volume artificial sweeteners as conventional sugar replacements. Nevertheless, the possibility of employing alternative natural sweeteners, such as Stevia, oligofructose, and isomaltulose, is now opening up, with the advantage of their providing some healthy benefits. In this section, we try to present the most recent and relevant contributions in this field, which are summarized in Table 1.

In a very comprehensive work, Struck et al. [27] summarize the state-of-the-art in the use of both natural and artificial high-intensity sweeteners, as well as fructans and polyols (as bulking agents) instead of traditional sugars in sweet baked products and their effects on the characteristics of these goods. The authors point out that this process can be a challenging issue, since it has both advantages and drawbacks. Sucrose as a main component in sweet bakery products provides sweetness but also contributes to numerous processing and product characteristics; on the other hand, intense sweeteners—although their sweetness clearly exceeds that of sucrose—do not contribute significantly to the body of the product, whereas the substitution of sucrose by bulking sweeteners may give rise to products with a similar body but a lack of flavor and/or taste.

Another interesting review was performed by Ghosh and Sudha [28]. This paper focuses on some recent studies performed on sucrose replacement with polyols in baked goods. Polyols are a group of reduced-calorie sweeteners, and can be considered as natural and nutritive sweeteners. In fact, they are a group of low-digestible carbohydrates which can be used instead of sucrose as sweeteners and also as bulking agents, since their sweetness is slightly lower than that of sucrose. They are present naturally in foods and come from plant products such as berries and fruits. They are available in syrups as well as in solid crystalline form and provide the baker with a versatile range of ingredients to increase the available portfolio of products. 

High-intensity sweeteners also include steviol glycosides. They are natural non-caloric substances extracted from *Stevia rebaudiana* Bertoni leaves which are increasingly used as sweeteners for a range of foodstuffs. In another interesting contribution, Karp et al. [23] tried to evaluate the effect of substituting sucrose with steviol glycosides on the quality properties of baked goods, such as muffins. Different parameters such as texture, color, and browning index were analyzed, with a sensory analysis also carried out. According to the study, a 25% addition of steviol sweetener (instead of sucrose) was the most suitable modification of the conventional formulation. The resulting muffins turned out to have a higher sensory attractiveness, as well as health-promoting qualities. On the other hand, it was also observed that a reduction in sucrose in excess of 50% negatively affected the quality of muffins and their sensory characteristics.

Zahn et al. [24] studied the possibility of utilizing steviol glycosides for the partial substitution of sucrose in bakery products. As an example, muffins were baked with an iso-sweet amount of rebaudioside A along with several fibers replacing 30% of sucrose. Color, texture and chemical analyses of the resulting products were performed, and their sensory profile was also evaluated. The results provided by a multivariate analysis of both instrumental and sensory data clearly show that a mixture of rebaudioside A with either polydextrose or inulin gives rise to products with similar features to those of a reference. These replacement substances significantly reduced energy and increased fiber content. The employment of apple fiber or wheat bran as a bulk replacer for sucrose resulted in products with a different crumb color and a wholemeal off-taste; nevertheless, partial sucrose replacement by oat, pea or wheat fiber, cellulose or maltodextrin resulted in an increased crumbliness and reduced elasticity.

Erythritol is a sugar alcohol (polyol) occurring naturally in some fruit and fermented foods. It is 60%–70% as sweet as sucrose yet it is almost noncaloric, hence its increasing importance within the food industry. Erythritol is widely used as sweetener in low-calorie bakery products and candies. Nevertheless, its production (unlike that of other polyols) is a challenging issue, since it cannot be chemically synthesized in a commercially worthwhile way; therefore, current research efforts are trying to improve both productivity and yield. A short review by Regnat, Mach, and Mach-Aigner [29] gives an overview of the attempts to improve erythritol production, as well as their development over time.

It is well known that the use of prebiotics has positive effects in stimulating a healthy intestinal tract. The human gut is unable to digest them, so, while they taste sweet, they are calorie free; that is why naturally sweet prebiotics can replace high-calorie sugars in food and drink products. On the other hand, the thermal stability of the product is sometimes improved, along with other textural, sensory, and physiological benefits. A comprehensive paper by Singla and Chakkaravarthi [30] provides an overview of the various prebiotics available from different sources and their applications in a great variety of sectors of the food industry, most notably bakery and confectionary sectors. The effects observed as a consequence of the addition of several prebiotics are also commented on. From this article, it can be inferred that the research in this field opens up new opportunities for the development of a range of natural sweeteners, which can be utilized to replace sugar in, for example, bakery products.

Liang and Were [22] examined the impact of several liquid sweeteners (including syrups and honey) and relative humidity on the chlorogenic acid-induced greening of sunflower butter cookies, which takes place under alkaline conditions. Doughs had a similar initial pH (7.5–7.7) which increased to 8.4–9.1 after baking. The results obtained showed that cookies enhanced with maple syrup had the highest moisture and greening, corresponding with the lowest free chlorogenic acid. According to the authors, a satisfactory correlation (r = 0.91) was obtained between the % greening and the chlorogenic–lysine adduct content. The results of this work show that undesirable greening can be inhibited by the use of an alternative liquid sweetener.

The research work of Martínez-Saez et al. [16] evaluated the utilization of spent coffee grounds (SCG) from instant coffee as a food ingredient and its application in bakery goods. Different evaluation assays of SCG were conducted; the results obtained showed that SCG (4% *w*/*w*) are a natural source of low glycaemic sugars, resistant to thermal food processing and the digestion process, and completely safe. Therefore, the authors incorporated SCG in biscuit formulations as a novelty. Low-calorie sweeteners and oligofructose were also included in the food formulations, and corresponding sensory tests of the biscuits were performed. This work resulted in innovative biscuits in accordance with consumers’ preferences, being straightforward, with high nutritional and sensorial quality and the potential to reduce the risk of diabetes and obesity.

There is also growing interest in low-intensity sweeteners such as oligosaccharides, partly due to their prebiotic status; that is why oligosaccharides from different sources (fungi, algae, bacteria, and others) have been widely utilized as food ingredients. The non-digestible oligosaccharides have played different roles as sweeteners, dietary fiber, or weight-controlling agents in confectioneries. On the other hand, functional oligosaccharides have turned out to be of great utility in dental caries’ prevention, as well as in the regulation of blood glucose in diabetics. Considering the importance of these compounds, Patel and Goyal [25] discuss their natural sources, types, and physiological properties, and describe recent synthesis, purification and analysis methods. Promising recent developments in this area are also remarked on, to facilitate their further exploitation.

The purpose of Tavera-Quiroz et al. [26] was to develop a crispy snack (baked snack from green apples) with the addition of isomalt and maltodextrin. Isomalt (a reduction product of isomaltulose) is often utilized in bakery goods as a noncariogenic nutritive sweetener. Its physical, chemical, and sensory properties were then assessed, followed by an evaluation of its stability during storage and after being conditioned at different relative humidities. The use of isomalt had a protective effect on the apple tissue at high temperatures and also preserved the ascorbic acid (E-300) added along the baking process. The isotherms obtained reflected a resistant behavior pattern in regions with low water activity (aw), but, in the case of aw > 0.7, the moisture content increased drastically.

Finally, Rodríguez, Magan and Medina [11] compared the influence of (a) three different Stevia-based sugar replacers (named S1–S3 by the authors), (b) sucrose alone, and (c) a combination of sucrose and S1 on: (1) humectant properties, (2) relative colonization rates of sponge cake slices at 0.90 aw by some major strains at 20 and 25 °C and (3) shelf-life periods in days prior to visible growth. From the results obtained, it can be concluded that S1, sucrose, and their combination were able to reach aw levels close to those of glucose and glycerol mixtures, whereas neither S2 nor S3 significantly reduced water activity levels. This work also performs a comprehensive study of the growth that occurred in all treated sponge cake slices at both temperatures. Finally, the authors conclude that care must be taken when substituting sucrose with low-calorie sugar replacers based on Stevia glycosides. They point out that different products may have variable humectant properties and bulking agents which could decrease the potential shelf-life of intermediate moisture confectionery goods.

## 3. Components to Replace Fats

Oils and fats added to bakery products play a very important role in their formulation. During the mixing and beating of the ingredients, fat (due to its surface-active properties) contributes to the incorporating and stabilizing of gas bubbles in the dough, and also prevents the excessive development of gluten proteins. Moreover, in the baked product, fats improve the final texture and increase its volume [31]. On the whole, butter, margarine, lard, cream, hydrogenated coconut oil, and sunseed or olive oils are used in these products; the proportion of the liquid and solid phase in each one of them determines the ability to stabilize gas bubbles [32].

Fats may represent up to 20% or 35% of the product, those containing a high level of saturated fatty acids (SFA, related to cardiovascular diseases and obesity) being common. On the other hand, trans fats are usually added to bakery products; these types of fats are obtained from a hydrogenation process of vegetable fats in such a way that they have similar structural properties to those of animal fats (essentially, a higher melting point). This process converts a liquid oil into a solid paste that improves the texture of many foodstuffs. Nevertheless, trans fatty acids are also related to the risk of heart diseases, since they increase low-density lipoprotein (LDL) cholesterol levels and reduce those of high-density lipoprotein (HDL); that is why their consumption must be kept low [33].

Due to these reasons, a better quality of the type of fat consumed is of paramount importance. Over the course of time, different ingredients have been utilized to replace fat in foods (Table 2), be they of lipid or protein nature, or even from carbohydrates [34]. 

The use of lipidic substitutes (often containing omega 3) involves modifying the ratio SFA:MUFA:PUFA (MUFA = monoinsaturated fatty acids; PUFA = polyinsaturated fatty acids). Sunseed oil, commonly utilized in sponges and muffins, has a 10%:29%:61% ratio, whereas that of margarine in cookies and croissants is 50%:33%:17% [35]. These authors also tried to increase the MUFA content and reduce the level of SFA; for this purpose, they evaluated the replacement of margarine and sunseed oil by oleic-rich sunseed oil in several products (cookies, croissants, muffins, and sponges). They found that these new formulations not only improved the lipidic profile but also reduced the total amount of fat, and thus their caloric value. Nevertheless, only cookies received a good sensorial score.

In this way, the change in the lipidic profile of bakery products using seed oils can be a good strategy to increase their nutritional profile. However, the addition of seed oils from flax (Linum usitatissimum), camelina (Camelina sativa), or garden cress (Lepidium sativum), with a high content of omega-3 fatty acids (mainly α-linoleic acid), gave rise to high oxidation ratios during the storage of sponges, unless microencapsulation techniques were used [36,55,56]. Rajiv et al. [37] point out the possibility of replacing up to 15% wheat flour with flax seeds with no significant changes in the peroxide value, during cookies’ storage in metallized polyester pouches at ambient conditions.

Chia seeds (Salvia hispanica L.) with a high oil content (30%–40%), mainly omega-3 (linolenic acid, 54%–67%) and omega-6 (linoleic acid, 12%–21%), as well as in protein (15%–25%) and fiber (18%–30%), aim at improving the nutritional profile of bakery products [43,57]. In this sense, many authors have used this seed in different formats.

According to Mesías et al. [38], the addition of ca. 10% chia flour to sponges improved the nutritional value of the product, but also the levels of acrylamide, hydroxy methyl furfural, and furfural; however, it reduces the shelf life of the product by accelerating the oxidation of lipids. Pizarro et al. [39] studied the replacement of wheat flour by chia flour (up to 30%) in the preparation of sponges, which resulted in a decrease in both specific volume and color parameters with increasing chia flour concentration. These negative effects were countered by the addition of hydrogenated vegetable fats, with an optimum formulation being obtained by 15 g chia flour and 20 g hydrogenated fat (total flour = 100 g). Coelho and de las Mercedes Salas-Mellado [40] optimized a bread formulation using chia flour or seeds, with a PUFA:SFA ratio of 3.1 and 3.9 (control bread had a ratio of 1.01).

Chia mucilage, obtained from seed hydration, can also act as fat replacer, thanks to its high water-absorption capacity and increase in dough viscosity [41]. The substitution of either 25% of fat in sponges [58] or 50% in sponges and biscuits [42,43], by dehydrated chia mucilage (by means of hot air or lyophilization) does not significantly modify the organoleptic characteristics of sponges, whereas the lipid profile improves. 

Some researchers have studied the influence—on dough and final product properties—of the replacement of fats traditionally used in bakery wares by hemicellulose-rich ingredients, such as green banana puree (GBP) [44]. The substitution of butter in different proportions (evaluated through sensory attributes) gave rise to changes in color, texture, odor, and taste of the sponges. It was feasible to substitute 25% of fat with GBP in pound cakes, as well as to reduce 20%–40% of sugar in low-fat cakes with GBP, with very little impact on acceptance and sensory characteristics. By-products of the fruit industry (rich in bioactive components) have also been studied as fat replacements, for instance, berry pomace in sponge cakes (30% substitution) [59], avocado purée in muffins [60] (50% substitution), and okra gum [61] (100% substitution) in chocolate bar cookies; products with a good sensorial acceptance were obtained in these cases. 

Several authors have evaluated the reduction in the fat content in sponges or muffins by using different replacers based on carbohydrates, such as xanthan gum [45], guar gum [46], hydroxypropyl methylcellulose [45,47,48] maltodextrins [46,48,49,50,51], dextrins [52], or inulin [53]. In general, this substitution affects the textural properties of the baked products, them being harder, and thus they have lower acceptance levels than control products.

## 4. Substitution of Proteins

Eggs are used in baking for several important functional properties, such as binding, leavening, tenderizing, volume, texture, stabilization, emulsification, foaming, coagulation, flavor, color, and food/nutritional value [62]. Nevertheless, eggs are considered one of the most allergenic foods, and that is why their possible substitution in bakery products has been extensively researched. Most commonly used commercial egg replacers use whey protein isolates, soy ingredients, wheat gluten, and different types of gums as the main functional components in the ingredient mixture in order to obtain specific properties in bakery products [62].

In addition to egg protein, most of the protein in bakery products comes from flour, which forms an elastic dough along with the water. Gluten proteins, mainly gliadin and gluteine, are also highly allergenic. Thus, gluten replacement in bakery products is a fundamental technological challenge, due to its essential structural binding properties. In recent years, numerous researchers have been working on the development of gluten-free products by the substitution of wheat floor with different gluten-free cereal flours such as those from rice [63,64,65], acorn [66,67], soybean [68], sorghum [69,70,71] or mixtures [72,73,74]. Other studies have evaluated wheat flour replacement by pseudo-cereals such as amaranth [75,76,77,78], quinoa [79,80,81], buckwheat [78,82,83,84] or legume flours [85,86,87,88,89]. 

On the other hand, when using gluten-free ingredients, substances that have the properties of gluten are frequently added as isolated proteins (from egg, legumes, or dairy products), hydrocolloids (alginate, guar and xanthan gums, carrageenan, carboxymethyl cellulose, hydroxypropyl methylcellulose), emulsifiers, or enzymes (proteases, transglutaminase, glucose oxidase, cyclodextrin glycosyl transferases and laccase) [86,89,90]. Table 3 summarizes some of the major contributions in the last few years in this field. 

## 5. Types of Fiber Used in Bakery and Other Potential Types of Fiber

Dietary fiber (DF) is the portion of plant-derived food that cannot be completely broken down by human digestive enzymes, and includes carbohydrate-based plant materials (wall polysaccharides, resistant starch and oligosaccharides) [91,92]. Depending on the solubility of the fiber, it can be classified as soluble or insoluble. Water-soluble fibers, which can be found in fruits (such as oranges, apples and grapefruit), legumes (dry beans, lentils and peas), vegetables, barley, oats and oat bran, absorb water during digestion, which increases stool bulk and may decrease blood cholesterol levels. In this case, compounds of high and medium molecular weight, such as soluble pentosans, soluble pectin, β-glucans, carragenans and gum Arabic, as well as low molecular weight fractions, such as inulin, fructooligosaccharides (FOS) galactoosaccharides (GOS), resistant maltrodextrins (RMD) and polydextrose, can be found [93]. Water-insoluble fibers, which are contained in fruits (edible peel or seeds), wholegrain products, bulgur wheat, vegetables, stone ground corn meal, cereals, bran, rolled oats and buckwheat and brown rice, do not change during digestion but stimulate peristaltic movements. They are composed of resistant starch (RS) or high and medium molecular weight fractions of cellulose, pentosans, pectin or lignin [93]. However, potential functional fiber for food may come not only from vegetables but also from animals (e.g., chitin and chitosan) and may be commercially produced (e.g., polydextrose, resistant starch, inulin and indigestible dextrins) [94] or even obtained from seaweeds [95]. Moreover, Pina-Pérez et al. [96] reviewed the most effective antimicrobial extracts from algae against foodborne pathogenic bacteria. They concluded that—as antioxidants and antimicrobials—algae have good prospects in response to the Horizon 2020 call for white label developments and innovation in sustainable food products.

It is widely known that a healthy diet based on a sufficient amount of dietary fiber may help to avoid the development of different ailments such as diabetes, hepatitis, and cardiovascular diseases, which are among the leading causes of death. Thus, dietary guidelines worldwide recommend consumption of wholegrain products as well as a daily intake of dietary fiber of 25–40 g for adults [97,98]. In fact, the European Prospective Investigation into Cancer and Nutrition (EPIC) has shown a 40% risk reduction of colorectal cancer when consuming more than 30 g of fiber/day [99,100]. However, in many western countries, diets are still often low in dietary fiber, because of the relatively low intake of edible plant tissues from vegetables, fruits and wholegrain cereal products. Hence, there is a worldwide need to develop new ingredients and foods with enhanced nutritional benefits and it is also necessary to know how properties of fiber are affected during food processing and how this can impact nutrient digestibility [92]. In this respect, the incorporation of dietary fiber into bakery products has been widely studied, since they are a staple food, which are regularly consumed by all social and age sectors. Recently, Sharma et al. [101] observed a decrease in hypocholesterolemic values in the serum of laboratory animals fed with milled muffins or cookies containing pumpkin flour.

Besides, there was a reduction in pathogens and a growth of lactic and bifidobacteria in the gastrointestinal tract of these animals. Nevertheless, the activity of all enzymes studied did not reach values comparable with those of healthy animals. However, it is important to bear in mind that the fortification of dietary fiber in food products negatively affects the product’s functional properties [102,103]. Thus, Foschia et al. [104] published a review explaining the effects of different dietary fibers (inulin, fructo-oligofructose, β-glucans, arabinoxylans and resistant starch) on the quality and nutritional aspects of common foods containing cereals, such as pasta, bread, muffins/cakes and extruded snacks. In the present study, an update of that review has been carried out, and the results are shown in Table 4. Furthermore, Kadam and Prabhasankar in 2010 [95] described the status and future projections of marine functional ingredients in bakery and pasta products. Seven years later, Roohinajad et al. [105] reported the different applications of seaweeds in the development of new food products with enhanced shelf life, quality, and health-related beneficial properties. The special attention given to the role of seaweeds in these bakery products is remarkable, since they contain a significant amount of soluble and insoluble polysaccharides, and have a potential function as dietary fiber, with a higher Water Holding Capacity (WHC) than cellulosic fibers. 

Moreover, for the celiac population, the needs of dietary fiber are especially important, since most of the cereal-based gluten-free products are usually made with starches and/or refined flours, and therefore low in fiber, so it is necessary to improve their nutritional quality [121]. In this regard, Talens et al. [110] compared the physico-chemical properties of a new orange fiber ingredient obtained by hot air coupled with microwave (HAD + MW) with a commercial citrus fiber and they also studied the differences between gluten-free muffins formulated with both orange fibers. According to the sensorial analysis, panelists preferred muffins with HAD + MW fiber to those with commercial fiber. Thereby, they concluded that the application of HAD + MW drying may be a new alternative for citrus by-product valorization and transformation into a fiber ingredient suitable for gluten-free baking. In this line, Różyło et al. [117] studied the influence of brown algae addition on the physical, antioxidant, and sensorial properties of gluten-free bread (GFB). They concluded that an acceptable GFB could be obtained by adding 2% or 4% of the algae.

## 6. Conclusions

Due to the evident relationship between diet and health, there is a growing interest in improving the nutritional profile of most food products, especially those with high sugar and fat contents. Bakery products are consumed by all sectors of the society regardless of age and income level. 

A major conclusion to be drawn in this field is that current research efforts are bearing fruit. In bakery products, traditional ingredients providing carbohydrates, proteins, and fats may be successfully replaced by other, healthier substances, without nutritional quality loss. The different approaches described in this review seem to corroborate this claim.

As recommended by international organizations, such as the WHO (World Health Organization), this paper demonstrates the feasibility of replacing critical ingredients in bakery products with other healthier substances—the resulting products still being acceptable for the consumer. Nevertheless, although all published articles are focused on the nutritional improvement caused by the replacement of some ingredients, there remains the problem of the combined effect of the simultaneous substitution of several of them. 

## Figures and Tables

**Table 1:** Major sugar replacers used in bakery products.

Bakery Product (s)	Substance (s) Proposed	Substitution Level	Remarks	Reference
Sunflower butter cookies	Different sweeteners	2% (*w*/*v*) concentration in all instances (maple syrup, xylitol, corn syrup, agave syrup, honey)	Influence of several sweeteners on greening of sunflower butter cookies	[22]
Biscuits	Low-calorie sweeteners and oligofructose	100% oligofructose	New biscuit formulations using low-calorie sweeteners	[16]
		70% maltitol – 30% stevia		
Muffins	Steviol glycosides	25% steviol sweetener	Quality effect of sugar replacement by steviol glycosides	[23]
Muffins	Steviol glycosides	50% stevianna or 50% inulin	The use of steviol glycosides as partial a replacement for sucrose	[24]
Different types	Functional oligosaccharides		Study of the properties of different functional oligosaccharides as potential sucrose replacers	[25]
Baked snack from green apples	Isomalt, maltodextrin	30% (*w*/*v*) isomalt and 30% (*w*/*v*) maltodextrin	The effect of using alternative noncariogenic nutritive sweeteners	[26]

**Table 2:** Different proposals for fat replacements in selected bakery products.

Fat Replacer	Products	Substitution Level	Results	References
High oleic sunflower oil	Cookies	100% margarine	Better nutritional properties	[35]
	Croissants	20% margarine	Only cookies maintained	
	Spanish muffins	100% sunflower oil	sensory acceptability and purchase intention	
	Spanish sponge cake	100% sunflower oil		
Garden cress (*Lepidium sativum*) seed oil or Microencapsulated garden cress oil powder	Biscuits	25% bakery shortening fat	Enhanced the nutritional quality of products with α-linolenic acid	[36]
			Increases the shelf-life over storage of biscuits with alpha-linolenic acid (ALA) microencapsulated	
Flax seed (Linum usitatissimum)	Cookies	Wheat flour with 0%, 5%, 10%, 15% and 20% flax seeds	Beyond 15% level of recovery growth factor (RGF) substitution adversely affected cookies quality	[37]
			Acceptable quality cookies with omega-3-fatty acid can be prepared by substituting 15% RGF	
Chia (Salvia hispanica L.) flour	Biscuits	Wheat flour with 0%, 5%, 10%, 15% and 20% chia flour	Nutritionally improved product, with higher amounts of protein, dietary fiber, antioxidants and polyunsaturated fatty acids	[38]
			Increased the formation of acrylamide, hydroxymethylfurfural (HMF) and furfural and promoters such as methylglyoxal	
Whole chia flour (*Salvia hispanica L.*) and hydrogenated vegetable fat	Pound Cakes	0–30 g chia flour/100 g flour mixture and 12–20 g hydrogenated vegetable fat/100 g flour mixture	The best technological results were in cakes containing up to 15 g whole chia flour (WCF)/100 g flour mixture and from 16 to 20 g hydrogenated vegetable fat (HVF)/100 g flour mixture	[39]
			Nutritionally enhanced, mainly in relation to the omega-3 content and omega-6/omega-3 ratio	
Chia seeds and chia flour	Bread	0–20 g chia flour/100 g wheat flour and 0–3 g hydrogenated vegetable fat/100 g wheat flour; 2–20 g chia seeds/100 wheat flour and 0–3 g hydrogenated vegetable fat/100 g wheat flour	Increased the ratio PUFA:SFA from 1.01 (control bread) to 3.1 (chia flour) and 3.9 (chia seeds)	[40]
			Decrease in the specific volume	
			In sensory evaluation, high levels of acceptability and purchase plans	
Chia mucilage gel	Cakes	25, 50, 75 and 100 g/100 g of vegetable fat by chia mucilage gel (CMG)	Formulations with up to 25/100 g of fat substitution presented similar technological characteristics to the control	[41]
			Levels superior to 25/100 g of fat substitution affects negatively color and texture	
Chia (Salvia hispanica L.) gel	Cakes	25%, 50% and 75% of oil	Decrease ratio of n-6 to n-3 fatty acids from 215.7 (control) to 13.2 (75% oil substitution)	[42]
			Cake weight was not statistically different from control at any substitution level	
			Cake volume decrease with substitution increase above 50%	
			Significant effect when replacing above 50% for acceptability, color, texture and taste	
Chia mucilage (CM) dryed	Bread and chocolate cakes	25%, 50%, 75% and 100% of vegetable fat	Reduction in caloric value	[43]
			The bread prepared with 75% showed a higher acceptability and greater purchase intent	
			For chocolate cakes, chia mucilage can replace up to 50% of fat without affecting the technological and physical characteristics	
Green banana puree	Pound cakes	0%–100% of fat	Increased firmness, springiness, luminosity, color saturation and hue angle in the crust of the product. The best results were obtained with 25% replacement	[44]
Oil/gel systems: sunflower or olive (47%)/HPMC or xanthan gum (2%)	Biscuits	100% of shortening at 18% fat	The biscuits prepared with either olive oil or sunflower oil and xanthan gum differed the most from the biscuit control. The biscuits formulated with either olive oil or sunflower oil and HPMC had the closest sensory properties to the shortening biscuits.	[45]
Maltodextrin and guar gum	Biscuits	Formulations with fat 10.5%–24.5%, maltodextrin 10.4%–24% and guar gum 0.1%–0.5%	Optimized product has 62.5% replacement of fat with maltodextrin and guar gum	[46]
Emulsions sunflower oil (51%), hydroxipropilme-thylcellulose (4 or 250 Pa s^−1^) (2%), water (47%)	Muffins		The emulsion muffins were significantly harder and had a lower sensory acceptability	[47]
Inulin, hydroxypropyl methylcellulose and maltodextrin	Pea cracker	0%, 25%, 50%, 75% and 100% of canola oil	Snack hardness increase and browning decrease as fat replacement level increased	[48]
			Snacks are accepted by consumers with 75% inulin or maltodextrin	
Corn fiber, maltodextrin and lupine extract	Biscuits	30% or 40% fat	Increased moisture content after baking; volume increase was lower and the firmness increased drastically.	[49]
			All changes highest when lupine extract was used	
Polydextrose (PD) and Simplesse^®^	Biscuits	10%–40%	PD is more suitable than Simplesse^®^	[50]
			PD can be used up to 30% to partially replace the fat without significantly affecting the sensorial properties	
			Reduction of 15.98% of energy and 30% less fat content	
Maltodextrin	Biscuits	Ratio maltodextrin:bakery fat (0:42 to 35:7)	Biscuits with 20 g replacement got the highest sensory score	[51]
N-Dulge (tapioca dextrin and tapioca starch)	Biscuits	10% and 20% of shortening	Increase hard and crumb with fat decreased by 10% shortening replacement achieved samples with good acceptability	[52]
Inulin	Short-dough biscuits	74.1%, 64.8% and 55.4% margarine and 0.9% and 18.3%		[53]
Emulsion filled gel (EFG) based on inulin and extra virgin olive oil	Shortbread cookies	50% and 100% of butter replaced by EFG	Cookies with EFG had thinner pore walls. Cookies with 50% EFG showed similar microstructure and fracture properties to control and were well accepted by consumers	[54]

**Table 3:** Selected alternatives for protein replacements in bakery products.

Protein Replaced/Alternative	Products	Substitution Level	Results	References
Whole egg/three commercial egg replacers (R1, R2 and R3)	Muffins	- 25% dry whole egg + 75% R1	At 100% replacement, none of the commercial egg replacers produced acceptable quality muffins. Partial replacement of egg changed moisture retention, bulk volume, color, texture and flavor. Some of these differences, were not detected by the sensory panelists	[62]
		- 25% dry whole egg + 15% R2		
		- 50% dry whole egg + 50% R3		
Wheat flour/rice, corn, soy flour	Gluten-free bread	Rice (100)	Breads made with rice, corn, and soy flours showed the best quality attributes: high specific volume, good crumb appearance, soft texture, and low staling rate. The addition of soy caused crumb softening and retarded bread staling	[63]
		Rice/Corn (50:50)		
		Rice/Soy (90:10)		
		Rice/Soy (80:20)		
		Corn/Soy (90:10)		
		Corn/Soy (80:20)		
		Rice/Corn/Soy (45:45:10)		
		Rice/Corn/Soy (40:40:20)		
Wheat flour/white, brown and germinated brown rice (GBR)	Sugar-snap cookies	30%, 50%, 70% and 100% of wheat flour	All cookies containing rice flours required significantly less force to compress than the wheat flour cookies. Softening effect was increased as the level of rice flour substitution increased. Cookies made with the GBR flour displayed inferior physical characteristics compared to those with wheat flour.	[64]
Wheat flour/hydrothermal treatment of rice or corn flours and field bean	Gluten-free bread	100% of wheat flour by ratio 2/1 (*w*/*w*) cereal/field bean semolina	Hydrothermal treatment of rice or corn flours increases the specific volume of breads and H/W ratio and decreases the hardness and chewiness	[65]
Wheat flour/rice flour or corn starch with acorn meal	gluten-free bread	100% of wheat flour by rice flour and corn starch (1:1) with acorn meal addition (5%, 15%, 25%)	Acorn-supplemented gluten free breads better met sensory preference in terms of color and nutritionally improved in terms of total phenolics. The specific volume of breads significantly decreased with increasing acorn addition, while crumb hardness increased	[66]
Wheat flour/acorn flour in biscuits		30 and 60 g, 100 g^−1^ on wheat flour basis.	Biscuits with acorn showed a higher content of phenolics, antioxidant activity and oxidative stability than control biscuits. They were also darker, larger, more voluminous and more friable than control biscuits.	[67]
Wheat flour/cornstarch and white sorghum	Gluten-free bread	100% of wheat flour by different cornstarch/sorghum flour ratio	The optimized recipe was 0.55 cornstarch/sorghum flour ratio	[69]
Wheat flour/sorghum flour	Gluten-free bread and cake	100% of wheat flour by dry heat sorghum flour at two temperatures	Heating the flour at 125 °C for 30 min produced bread and cakes with the highest specific volume and the most cells per slice area. Cake and bread made from this heat treatment were more acceptable than the controls in consumer testing	[70]
Wheat flour/sorghum flour	Gluten free biscuits	100% of wheat flour by sorghum flour of different particle sizes	The hardness was higher in biscuits prepared from flour of particle size 152, 104 and 75 μm compared to 251 μm and 180 μm. L* and b* parameters were higher in hammer-milled flour and a* and whiteness index were higher in traditionally milled flour. Acceptability was higher in biscuits prepared from the traditionally milled flour of particle sizes.	[71]
Wheat flour/amaranth, buckwheat, corn, chickpea, millet and quinoa flour (MF) and rice flour (RF)	Gluten-free bread	100% of wheat flour by ratio FM:RF of 1:1, 3:7 and 7:3	The most positive impact was with the presence of buckwheat flour. Millet and corn flour negatively impacted rice dough behavior, resulting in bread of unacceptable quality	[72]
Wheat flour/rice, maize, sorghum and pearl millet flour	Cookies	100% of wheat flour by flour combinations (50:50)	All the blends of flour significantly improved pasting qualities, functional properties, sensory qualities and nutritional values	[73]
Wheat flour (W)/oats (O) and finger millet (M)	Cookies	Ratios O:M:W: 10:10:80, 20:20:60, 30:30:40 and 40:40:20	Oat and finger millet flour addition significantly improved the dietary fiber content, protein content and crude fat content.	[74]
Wheat flour/amaranth flour	Cookies	20%, 40%, 60%, 80%, and 100%	Hardness of cookies decreased with the addition of amaranth flour. Amaranth cookies with up to 60% were sensory acceptable	[75]
Wheat flour/raw and germinated amaranth grain flour	Cookies	100% of wheat flour	Raw amaranth flour cookies showed the highest spread ratio, followed by germinated amaranth flour. Germinated amaranth cookies exhibited highest antioxidant activity and total dietary fiber. Acceptable quality and improved nutrition found in gluten-free cookies with germinated amaranth flour.	[76]
Wheat flour/composite amaranth-oat flour	Cookies	100% of wheat flour by amaranth flour or ratio 3:1 amaranth-oat flour	Amaranth and its composites improved water-holding capacities. Amaranth-oat cookies were acceptable in color, flavor and texture with no significant differences in sensory qualities. They also had enhanced nutritional value	[77]
Whole wheat flour, amaranth flour and buckwheat flour/chickpea	Cookies	0%, 20%, 40%, 60%, 80% and 100% of chickpea	Optimal levels of chickpea addition were 20%–40% in wheat cookies and 60%–80% for amaranth and buckwheat cookies	[78]
Wheat flour/quinoa flour, quinoa flakes and corn starch	Cookies	100% of wheat flour by composite of quinoa flour, quinoa flakes and corn starch	Optimized formulation: 30% quinoa flour, 25% quinoa flakes and 45% corn starch	[79]
Wheat flour/quinoa flour	Cookies	Wheat flour by 10%, 20% and 30% quinoa flour	Partial replacement of wheat flour by quinoa flour (up to 30%) increased the nutritional value of the cookies without changing the sensory characteristics	[81]
Wheat flour/buckwheat flour	Cookies	Wheat flour by 0%, 20%, 40%, 60%, 80% and 100% buckwheat flour	Increased the antioxidant properties of blended flour and metal chelating properties, hardness and spread ratio decreased	[82]
Rice flour/buckwheat flour	Cookies	Rice flour by 10%, 20% and 30 % buckwheat flour	Higher mineral content and total phenolic content. Cookies containing 20% light buckwheat flour had the most acceptable sensory properties	[83]
Rice flour/buckwheat flour and carboxymethyl cellulose	Cookies	Rice flour by 10%, 20% and 30% buckwheat flour. Formulation containing 20% of buckwheat flour without the addition of 0,69% CMC	Addition of CMC increased dough tenacity and resistance to deformation. Buckwheat addition decreased hardness and fracturability of cookies, as well as the overall acceptability	[84]
Wheat flour/chickpea flour, pea isolate, carob germ flour or soya flour	Gluten-free bread	100% of wheat flour	Chickpea bread showed the softest crumb, the best physico-chemical characteristics and, in general, good sensory properties	[85]
Wheat flour/jering seed flour	Cookies	0%, 5%, 10%, 15%, 20% and 100% of wheat flour	Cookies had higher protein, fiber and ash. Changes in optical properties with jering addition. Most acceptable sensory quality with 10% replacement	[86]
Wheat flour/lentil flour	Layer and sponge cakes	50% and 100% of wheat flour	Lentil flours reduced the density of layer-cake batter but increased the density of sponge-cake batter. Adding lentil flour reduced layer-cake volume, symmetry index, cohesiveness and springiness and increased hardness. In sponge cakes, substitution of wheat flour with lentil flour gave rise to harder and less cohesive cakes	[89]
Durum wheat and semolina/Apulian Black Chickpea wholemeal flour	Bread, focaccia and pizza crust	DW (durum wheat re-milled semolina) BC (product prepared by using a composite meal containing 60/100 g of durum wheat re-milled semolina and 40/100 g of Apulian black chickpea wholemeal flour)	Apulian black chickpeas to durum wheat re-milled semolina caused a decrease in the bread-making but there was a nutritional improvement in terms of higher contents of fiber and proteins	[88]

**Table 4:** Types of fiber used in bakery along with potential replacements.

Type of Fiber	Aim	Level of Replacement in Bakery/Bread/Cake	Properties in Dough	Properties in Final Product	References
**DERIVED FROM FRUITS, VEGETABLES AND GRAINS**					
Coffee silverskin	Determine the influence of coffee silverskin as a fat substitution in cakes treated (WTCS) or not (UTCS) with water to reduce bitterness	Cakes were formulated with 0%, 20%, 25% and 30% replacement of fat with coffee silverskin	Not reported	WTCS cakes were more similar to the control cake and they were found to be preferable to UTCS. Substitution of fat in cakes up to 30% by WTCS is feasible.	[106]
Soluble cocoa fiber	Assess the effects of a soluble cocoa fiber as a fat replacer in chocolate muffins and their batters	Part of the oil ingredient (25%, 50% and 75%) was replaced by soluble cocoa fiber, and control sample to which cocoa powder was added, for comparison purposes.	Fiber increased the consistency and decreased the flow index, indicating a more entangled structure	Cocoa fiber gave muffins higher moisture and a more tender and crumbly texture, also reducing hardening during storage. However, there was a loss of height, bitter taste and surface stickiness.	[107]
Spent coffee grounds (SCG) along with low-calorie sweeteners and oligofructose	Evaluate the use of SCG from instant coffee as a food ingredient and its application in bakery	SCG added to the biscuits ranged 3.5%–4.4%, to achieve the nutritional claims “source of fiber” (≥3 g fiber/100 g biscuit) and “high fiber content” (≥6 g fiber/100 g biscuit)	Not reported	SCG are natural source of antioxidant insoluble dietary fiber, proteins, essential amino acids and low glycaemic sugars. SCG (4% *w*/*w*) can be used directly as ingredient without affecting the conventional food preparation and the final product	[16]
Flour isolated from green bananas (GB)	Influence of wheat flour replacement by GB flour, with three sizes, on the nutritional, physical and sensory properties of layer and sponge cakes.	Wheat replacement: 15% and 30% by GB flour	BG flour reduced batter density for both layer and sponge cakes	A plausible 30% replacement of banana flour in layer cakes is demonstrated, finding only a small decline in the sensory perception. However, sponge cakes were worsened with banana flours, especially with a higher size.	[108]
Wholegrain concentrate (WGC) (6% of total dietary fiber in the end product)	Develop healthy fiber-enriched and wholegrain bread products with sensory attributes similar to white bread.	3000 g flour or 3000 g wholemeal flour or a combination of 2670 g refined flour and 330 g WGC	Dough stickiness tended to be increased when refined flour was replaced by wholemeal flour	15% WGC in bread roll increased fiber, Fe, Mg, Zn and folate. For tin breads, about 24% WGC was required to obtain this nutritional quality	[109]
Orange fiber (OF)	Compare twi gluten-free muffins formulated with two different OF: obtained by hot air coupled with microwave drying (HAD + MW) of orange peels or commercially available.	Water (43%), sugar (18%), sunflower oil (11%), whole egg (7%), skim milk powder (6%), corn starch (5%), citrus fiber (4.5%), rice flour (4%), leavening agents (1%), salt (0.5%).	Total dietary fiber, water retention capacity, viscosity and viscoelastic properties (G’ and G”) were higher for HAD + MW fiber	Panellists preferred HAD + MW muffins due to their attractive color, flavor, texture and chewiness.	[110]
Chia seed mucilage (CM)	Extract CM, dry it at 50 ºC, or lyophilize, and evaluate the effects of its incorporation on the technological quality of breads and pound cakes	Fat in bread and cakes was replaced by CM at 25%, 50%, 75% and 100%.	Not reported	Breads and chocolate cakes made with CM can replace up to 50% of fat without affecting the technological and physical characteristics	[43]
Chia seed flour	Test the capacity of chia seed flour to improve the bread-making process of fiber-rich dough and product properties.	Seven formulas: one with wheat flour, two substituting 13% and 23% (d.b.) of wheat flour with bran, and the last two were combined in turn with chia substituting 5% and 10% (d.b.) of their wheat flour	Chia led to an increase in the gas retention of dough with 13% of bran	The 13% bran/5% chia formula generated breads with 12% fiber content had no differences in specific volume and similar hardness compared to the refined wheat ones. The same sensory scores were shown with respect to the wholemeal formula without chia flour.	[111]
Sorghum flour	Evaluate starch, dietary fiber and mineral content of cookies developed from 12 sorghum cultivars.	Wheat flour was totally replaced by sorghum flour from 12 different cultivars	Not reported	Sorghum cookies had higher dietary fiber content than control. Three selective cultivars (CSH 23, CSH 13R and CSV 18R) had the best acceptability and were rich in nutritional qualities	[71]
Soybean meal (SBM)	Effect of dry-heating or fermentation by Saccharomyces cerevisiae on (SBM) composition	95% of wheat flour was replaced by SBM	Not reported	SBM biscuits showed adequate technological properties, improved nutritional and functional qualities and good sensory acceptance, whereas fermented SBM biscuits showed low sensory scores.	[112]
**COMMERCIALLY PRODUCED CARBOHYDRATES (OLIGOFRUCTOSE, INULIN, POLYDEXTROSE, RESISTANT STARCH)**					
Chicory fructans: Inulin Instant (inulin), Fibruline DS2 (inulin) and Fibrulose F97 (oligofructose)	Effect on bread-making of functional bakery goods, which justifies a prebiotic claim.	Wheat flour half-white with addition of 5%, 10%, 15% and 20% of inulin (Fibruline DS2) (% basis flour)	Upon addition of inulin, significant decrease in water absorption. Decreasing trend of dough machinability.	Breads had loaf volume reduced, were underdeveloped, with shriveled crust and irregular pores. Crumbs become harder and darker, but pleasant taste. Acceptability limit: 5% Fibruline DS2.	[94]
Inulin (Frutafit HD^®^)	Improve cake quality by adding an emulsifier mix and a lipase into cake batters in which fat was replaced with inulin	Fat-replaced (0%, 50% and 70%) with inulin (0, 7.5 and 10/100 g of flour, respectively). Dispersion inulin-to-water, 1:2 was added as a fat mimetic	Lipase reduces the degree of system structuring, whereas emulsifier increases batter consistency.	Good-quality cakes with 50% and 70% fat replacement can be obtained using lipase or emulsifier at low levels	[113]
Hydroxypropyl methylcellulose (HPMC) and inulin (Frutafit HD^®^)	Study the effect of partial fat replacement with inulin and HPMC in biscuits. Texture was studied by fracture and sound emission measurement.	15 and 30/100 g of the fat has been replaced by two different carbohydrate-based fat replacers (inulin and HPMC)	Not reported	Biscuits with Inulin and (HPMC) were harder and with higher sound emissions for control. 15/100 g with inulin or HPMC provided acceptable biscuits, but a higher replacement decreased the acceptability.	[114]
Partially hydrolyzed guar gum (PHGG)	Effect of PHGG and water on physical and sensory properties of bread	Refined wheat flour was mixed with PHGG at 1%, 1.59%, 3.0%, 4.41% and 5.0%	PHGG increased the dough strength	PHGG improved textural properties of bread	[103]
**DERIVED FROM ALGAE**					
Microalgae *Chlorella vulgaris* (CV)	Addition of CV in a wheat flour to evaluate dough rheology and bread texture	Microalgae contents from 1.0 to 5.0 g per 100 g of wheat flour were tested	Up to 3.0 g of CV: positive impact on dough rheology, viscoelastic properties and gluten network strengthening	up to 3.0 g CV/100 g WF addition resulted in breads with an interesting appearance, but with a higher CV, global aspect was worsened.	[115]
*Fucus vesiculosus* seaweed powder (FV)	Effect FV addition up to 8% on wheat flour dough and bread properties	2%, 4%, 6%, and 8% of seaweed powder (flour basis, f.b.)	FV raised elongation dough viscosity and consistency index	A maximum of 4% FV could be added, without impairing the density and crumb texture of enriched bread	[116]
Brown algae (BA)	Influence of BA on physical, antioxidant, and sensorial properties in gluten-free bread	2%, 4%, 6%, 8%, and 10% of the total flour content	Not reported	A larger volume was obtained using 4% of algae. Lightness and yellowness of breadcrumb decreased with the addition of BA.	[117]
**DERIVED FROM ANIMALS**					
Chitosan	Effect of chitosan on the sensory properties and the shelf-life of cupcake	Cupcakes with shrimp shells chitosan concentrations 0%, 0.5%, 1%, 1.5%, 2% and 2.5%	Not reported	1.5% of improved sensory properties and prolonged the shelf life of cupcakes.	[118]
Chitosan and chitosan oligosaccharides	Investigate the effects of chitosan oligosaccharides and chitosan on the rate of staling and the properties of bread crumb and crust.	2.18% of batter formulation was composed by chitosan or different types of chitosan oligosaccharides	Not reported	Chitosan oligosaccharides and low molecular weight chitosan increased bread crumb staling rate to a much lesser extent than middle molecular weight chitosan	[119]
Chitosan	Effect of chitosan on acrylamide and HMF in model systems and in biscuit and crust models.	Appropriate amounts of Asn and Glc dissolved in 1% chitosan solution. In biscuits, water was replaced with 0.5% chitosan in 1% formic acid.	No reported	Chitosan did not significantly affect the formation of acrylamide in biscuit and crust models during heating.	[120]
